# Expanding the Discussion on Internet Addiction and Suicide Risk in Adolescents

**DOI:** 10.2196/77290

**Published:** 2025-06-25

**Authors:** Sasha Zaki

**Affiliations:** 1 Faculty of Medicine, Jinnah Sindh Medical University, Rafiqui H.J Shaheed Road, Karachi, 75510, Pakistan, 92 02199205185

**Keywords:** internet addiction, suicide risk, suicide attempts, adolescents, pathological internet use, risk factors, cohort study

I have read with immense interest the article published by Li and colleagues [1] entitled “The Association Between Internet Addiction and the Risk of Suicide Attempts in Chinese Adolescents Aged 11-17 Years: Prospective Cohort Study.” The authors’ efforts to explore the association between internet addiction and suicide attempts among Chinese adolescents are commendable, as they address mental health issues in the landscape of increasing internet use among youth. However, the authors are advised to comment on a few concerns.

The adolescents completed a self-administered questionnaire on their parents’ phones via WeChat. A study by Gearing et al [2] found a higher degree of community-level stigma toward an adolescent participant versus an adult regarding suicide in the Chinese context. This is significant, as adolescents may have withheld honest responses to sensitive topics like suicidality out of concern that their parents might access their answers.

Moreover, it would have been beneficial to determine how the adolescents engaged with the internet. Past research suggests that certain activities are associated with an increased risk of suicidality. For instance, a Taiwanese study demonstrated that some internet activities, such as video gaming, chatting, and gambling, increased suicide risk compared to other activities, such as watching news [3]. Further categorization of adolescents based on different internet activities would provide a more nuanced understanding of how internet addiction and different online behaviors correlate to suicide risk. Additionally, it could identify “high-risk activities” and determine which behaviors contribute more significantly to suicidality.
